# Evaluation of an FE Model for the Design of a Complex Thin-Wall CFRP Structure for a Scientific Instrument

**DOI:** 10.3390/ma12030489

**Published:** 2019-02-05

**Authors:** Enrique Casarejos, Jose C. Riol, Jose A. Lopez-Campos, Abraham Segade, Jose A. Vilan

**Affiliations:** Department Mechanical Engineering, University of Vigo, E-36310 Vigo, Spain; jriol@uvigo.es (J.C.R.); joseangellopezcampos@uvigo.es (J.A.L.-C.); asegade@uvigo.es (A.S.); jvilan@uvigo.es (J.A.V.)

**Keywords:** composite, CFRP, thin-wall, finite element model, contact problem

## Abstract

In this paper, the reliability of a finite element (FE) model including carbon-fibre reinforced plastics (CFRPs) is evaluated for a case of a complex thin-wall honeycomb structure designed for a scientific instrument, such as a calorimeter. Mechanical calculations were performed using FE models including CFRPs, which required a specific definition to describe the micro-mechanical behaviour of the orthotropic materials coupled to homogeneous ones. There are well-known commercial software packages used as powerful tools for analyzing structures; however, for complex (many-parts) structures, the models become largely time consuming for both definition and calculation, which limits the appropriate feedback for the structure’s design. This study introduces a method to reduce a highly nonlinear model, including CFRPs, into a robust, simplified and realistic FE model capable of describing the deformations of the structure with known uncertainties. Therefore, to calculate the deviations of our model, displacement measurements in a reduced mechanical setup were performed, and then a variety of FE models were studied with the objective to find the simplest model with reliable results. The approach developed in this work leads to concluding that the deformations evaluated, including the uncertainties, were below the actual production tolerances, which makes the proposed model a successful tool for the designing process. Ultimately, this study serves as a future reference for complex projects requiring intensive mechanical evaluations for designing decisions.

## 1. Introduction

The deployment of reinforced plastics for structural components is a continuously growing option for many industrial fields. Either carbon, glass, or kevlar fibre reinforced plastics provide materials with large strength-to-mass ratios. The success of the application of these materials is linked to the efforts for developing models and calculation tools. The specialized literature is ample, e.g., the recent reference books [1,2], papers dedicated to laminated carbon-fibre reinforced plastics (CFRPs) [3], honeycomb cells [4] and large deformations of layered materials [5].

This research presents a study case of a scientific instrument requiring an important mechanical structure. Many types of instruments typically demand light structures with the requisite of being a robust and safe mechanical frame, and usually also providing an accurate and tight positioning of the critical active elements. An ideal instrument should have a maximal active volume free of structural parts since more structural parts have less sensitivity and more additional side effects. Honeycomb like structures, made of thin-wall parts, can provide excellent solutions. Applying to this structure, CFRP materials may be a very appropriate choice. Instruments with these selections can be found in new generation large telescopes [6], spatial applications [7,8] and particle physics instruments [9,10,11,12].

Scientific instrument design requires the analysis of demands from rather different sources. The physics codes must evaluate the adequacy of sensor volume, shape, position, and segmentation for maximum efficiency for the instrument goals. The mechanical design has to be adapted to the sensor needs, and calculations evaluate the structural requirements. The structure deformations influencing the location and integrity of the sensors require re-designing stiffer parts, and finer adjustments of the mechanical system would provide an optimal sensor orientation.

Despite the integration of CFRP pre- and post-processors in commercial software packages dedicated to finite element (FE) models, the definition quality of the orthotropic materials may be limited due to the implementation of the micro-mechanical models. It is also common that many and diverse CFRP fabrics in the market suffer from the scarce experimental data available to correctly describe the material. If the structure is complex and the number of parts is large, any FE model becomes largely time consuming for both its definition and its calculation. In this paper, an effective and robust FE model was used as a design tool, which has been developed and described along the project using a methodology that can be deployed for similar complex structural cases.

With the objective to show the calculation of a complete instrument structure to better understand deformations, and be able to correctly adjust the orientation of the sensors, this paper is organised as follows: in Section 2, the instrument is described. In Section 3, the mechanical test bench is presented to obtain the reference data. In Section 4, the settings used in the FE models implemented for the study are discussed. In Section 5, the results related to the simulation models and experimental setups are compared and the best FE model selected. In Section 6, the best model is applied to the complete structure of the instrument and the results presented, and, finally, in Section 7, the conclusions of this work are stated.

## 2. The CALIFA-FAIR Calorimeter: A Case Reference

The CALorimeter for the In Flight detection of gamma-rays and light charged pArticles (CALIFA) is an instrument developed for experiments in the international research facility FAIR (Germany), and under R&D [13,14] involving 12 different European institutes.

CALIFA is a detector for nucleus-to-nucleus collisions dedicated to particle physics studies. Surrounding the collision point, it is necessary an adequate segmentation into single sensors to fulfill the required detector sensitivity. Sensors are prismatic bodies made of radiation-sensitive scintillating crystals with lengths (between 170 and 220 mm) and shapes depending on their location, with an average volume of 147 cm^3^, and with glued opto-electronic circuit boards at one end. For the correct working of the instrument, it is necessary to define the positions of 1952 individual sensors with a total weight of about 1300 kg, and also to provide the right shape for the instrument.

As mentioned in the Introduction, an adequate honeycomb structure was used as mechanical support [15] to hold the sensors (crystals), to conform to the shape around the centre, and to position the crystals with enough stiffness to guarantee the orientation of all the individual sensors. The honeycomb walls between sensors have to be as thin as possible to reduce to a minimum the interactions of the particles and radiation through the matter. This effect is ruled by the atomic number of the material; therefore, carbon-compounds are always preferred to typical light metals.

### Construction of the CALIFA Calorimeter

**Figure 1:**
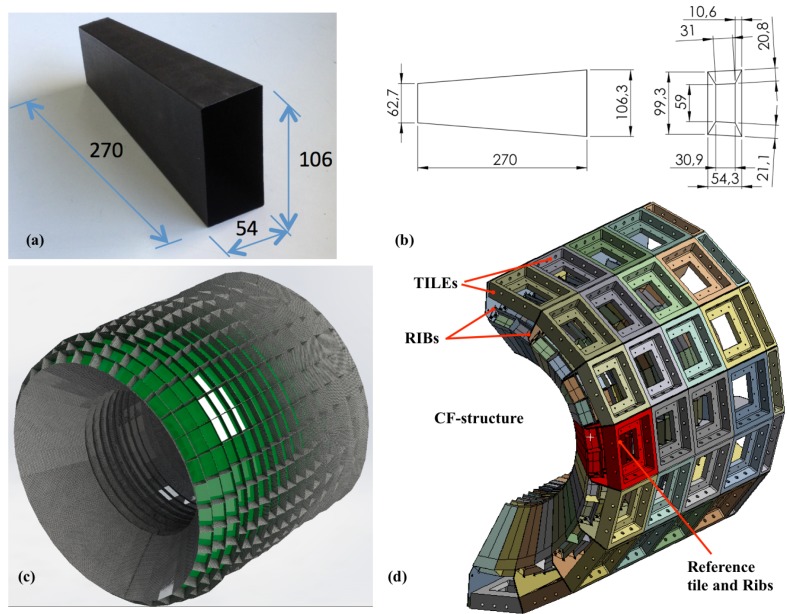
(**a**,**b**) picture and drawing of one CF-part (in mm); (**c**) drawing of the honeycomb CF-structure. The sixteen rings around the opening are built with 32 prismatic parts each; (**d**) cover structure built with 64 tiles for supporting the CFRP structure. Only one half is shown.

In this study, a CFRP fabric was used for the construction of the honeycomb structure (*CF-structure*), which was built with 512 CFRP thin-wall parts of 0.3 mm each. Each CF-part was a hollow prismatic box with lengths that range between 210 and 270 mm (Figure 1a,b), and were glued together with the neighbouring parts [15]. In addition, these CF-parts with adapted shapes were distributed in sixteen rings around an opening of 600 mm diameter, each ring built with 32 CF-parts (see Figure 1c). The CF-structure had an envelope of diameter 1060 mm and a length of 990 mm. The weight ratio of the structure with respect to the total mass resulted in a value as low as 0.7%.

**Figure 2:**
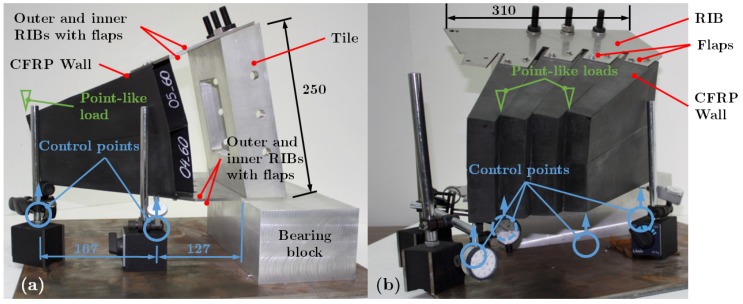
Pictures of the mechanical test bench. (**a**) setup with two CF-parts and two control points; (**b**) setup with eight CF-parts and three control points (one control point on the front was removed for this picture).

**Figure 3:**
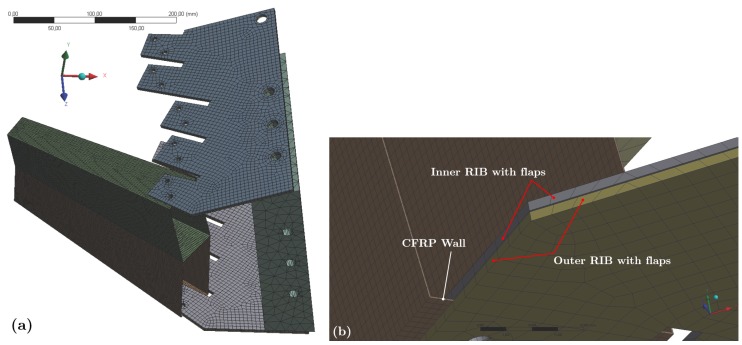
Drawings showing the mesh model used for the FE models. (**a**) setup with two CF-parts; (**b**) a detailed view of the rib-flap region where the CF-part is fastened.

Since sensors require a light- and gas-tight enclosure, a cylindrical cover around the CF-structure was the natural shape solution. This cover was designed with 64 similar parts called *tiles*; they were assembled in a regular pattern with an outer diameter of 1200 mm (Figure 1c). There were parts called *ribs* to hold the inner CF-structure as well as to connect with the cover. The ribs were 96 comb-like shaped plates with flaps. The reference tile and ribs for further calculations are marked in red in Figure 1d, and correspond to those in Figure 2. Each pair of ribs grabbed between their outer and inner flaps the walls of the CF-structure (Figure 2a and for more detail Figure 3b). The ribs were fastened in between the tiles of the cover providing the support of the inner CF-structure (Figure 1c). The cover is a very stiff and robust fastened assembly, which can be mounted on a gantry or equivalent external frame structure for the ultimate placement of the instrument [15].

The assembly of the CF-structure contains 512 CF-parts, which require being defined wall by wall in any FE model to specify the orientation of the fibre layer-by-layer of the CFRP because of the orthotropic nature of the CFRP material. Additionally, there are hundreds of metallic parts, and therefore thousands of contacts between the CF-parts, ribs, tiles, fasteners, and sensors inside the structure. This calorimeter was designed to be split in two equal halves by the vertical mid-plane (Figure 1d). This symmetry allows the calculation of only one-half of the structure but does not change the scale of the problem. Since the overall design required the evaluation of many different aspects in a feedback process with Physics simulations, there was a need to develop an effective and reliable FE model for the structure although reducing as much as possible its complexity for both definition and calculation.

A FE model was built as realistic as possible while preserving an effective definition for design. This study was based on selected definitions according to the results of benchmarking tests previously done on a singular part of the structure, and then an evaluation was conducted from a collection of FE models with different material and contact definitions. All the results were crosschecked with the reference data to get the deviation values and to allow the evaluation and analysis of the model performance and reliability.

## 3. Mechanical Tests Setup and Measurements to Obtain the Reference Data

Mechanical tests were performed in a robust setup, providing simple and easy to interpret data to compare the results to the FE models. The setups were mounted with different CF-parts to ensure that the use of specific parts made no difference to the results.

Two different setups were mounted with two and eight CF-parts, respectively, with dimensions as shown in Figure 2. The two configurations provided results with an important scale factor (400%) in order to study the whole structure from these reduced sets. The CF-parts were clamped firmly in between the flaps of the ribs at both sides, and fastened by bolts. The ribs, 2 mm thickness stainless steel, were fastened to a tile and a bearing block with bolts, being the same mounting system as for the whole structure.

Measuring deflection in the most unfavourable direction, where gravity acts on the most perpendicular rib of a tile corresponding to the red color tile in Figure 1d, ensures the appropriate performance of the structure. The lower wall of the CF-parts was set in the horizontal plane. Dial gauges (*Mitutoyo Corporation*, Kawasaki, Japan, 0.005 mm resolution) were firmly placed at the base plate to monitor the deflection of points located in that horizontal plane and away from the bearing region. Each load test was repeated several times to average the values, and check that both the displacement and the reference values were the same each time within an uncertainty limit value of 0.015 mm. Then, the control points (Figure 2) were measured at the flaps of the ribs as well as measured at the corners of the CF-part in that horizontal plane since it is the most compromised position for its farther (maximum) distance to the bearing region.

### 3.1. Setup with Two CF-Parts

For the setup with two CF-parts, a *finger like stick* was applied to input a vertical load up to a maximum of 13.5 N —a limited value to avoid compromising the integrity of the walls. It was also checked that when the load applied had a tiny component off the vertical, this one caused a deviation within the displacement uncertainty.

The displacement was measured at two points at the horizontal plane (Figure 2a), and found a linear dependency on the load values within the uncertainty. The response of the models reproduced that trend, thus only the maximum displacement was of interest. It was also verified that deflection in other directions different than the vertical one did not appreciably disturbed either the setup or the models; therefore, only the vertical direction was monitored at the two points, one at the corner of the CF-part and the other one at the corner of the rib.

Furthermore, due to the weight caused by filling the CF-parts with the prismatic sensors, a *realistic loading* of the structure was defined. The material used had a rather high density of 4.5 g/cm^3^, and the resulting weight was up to 2920 g per CF-part with an average value of 2645 g in comparison to the weight of the CF-part which was between 23 and 28 g. To describe this actual load situation, a second measurement was done by introducing steel blocks with a shape, weight, and centre of mass equal to the actual sensors inside the CF-parts.

### 3.2. Setup with Eight CF-Parts

For the same tile, the assembly with eight CF-parts was prepared (Figure 2b); this assembly resulted rather rigid and compact. It was not possible to load it with blocks without dismounting all parts, and, therefore, only point-like loads were applied. To produce the deflection, a load up to 40 N (again limited by the wall integrity) was applied at two different locations (green triangles in Figure 2b). The four points monitored with the control gauges at the horizontal plane were placed with two at the corners of the CF-parts and the other two at the corners of the rib; and this can be appreciated in Figure 2b, where the right front control gauge at the corner has been removed for a better view.

## 4. Definition and Analysis of the FE Models

All numerical calculations of FE models, as well as pre- and post-processing, were done with the software ANSYS (v15, ANSYS Inc., Canonsburg, PA 15317, USA), and using the composite pre-processing package (ACP). This package allows defining the CFRP layup and also couples the CFRP properties with those of the rest of materials within the solver. The ANSYS software is a widely deployed and well established tool for mechanical analysis including CFRP materials [12,16,17,18]. The orthotropic character of the CF-parts and the frictional contacts resulted in a nonlinear model which was solved by using the Newton–Raphson methods.

The FE models require defining the material properties, mesh, contacts, loads, and support conditions described in the following sections.

### 4.1. Material Properties

The walls of the CF-parts were made with two layers of epoxy-CFRP prepreg 1 K plain weave fabric of 0.15 mm thickness, with resin content of 40% (weight) produced by Cytec Industries Inc., (Woodland Park, NJ 07424, USA). The layers were set with different relative orientations, varying from face to face due to the wrapping of the fabric around the mould. The material properties depend on the fibre and matrix components, yarn size, 2D knitting of the yarns, and matrix impregnation. Manufacturers usually offer measured data for fabrics which are largely deployed in the industry. Therefore, final users have to perform their own tests according to appropriate standards [19,20,21,22,23] for other fabrics. This task is very time consuming and expensive.

However, micro-mechanical models [24] can provide estimates of the properties of the fabrics, sometimes with excellent predictive capabilities [25]. Authors in [26,27] developed a model for plain fabrics including the properties of the out-of-plane direction. The mechanical parameters defined according to the model described in [27] for the chosen fabric are listed in Table 1. Besides the material properties for the CFRP pre-processing, it was also necessary to introduce the fabric woven type, thickness, layers, and the orientation of its fibres in each layer of each face of the parts. The model of the CF-structure was made of 512 parts with five faces and two layers each. Therefore, this method can be used for small models like the two CF-parts setup, but, for larger models, the characterization of the orthotropic materials with such pre-processors would be largely time consuming.

Orthotropic materials may be useful to counteract asymmetrical load patterns. In the case of this instrument, the load demand is isotropic, the CF-parts being located at any orientation in the structure, and the sensor weight acting as load. The selection of the fabric and layering design of CFRP parts was done to optimize the performance of the CF-part under the actual tri-axial loads. The double layer of plain fabric was selected to effectively achieve an isotropic behaviour [28]. Based on that assumption, the possibility to define an ad hoc isotropic material for the definition of the CFRP actual behaviour was studied. The properties of this *virtual* material were defined with the same Young’s modulus value, and the shear modulus was adapted to reproduce the measured results. The value of the Poisson’s ratio, linked to the previous parameters, resulted in a generic and reasonable value, causing no extra side-effects on the results. The values of the properties for this virtual material are listed in Table 2 as CFRP*.

Typical values found in literature for the metallic parts [29], ribs (steel AISI-304) and tiles (aluminium 5083) are also listed in the Table 2.

### 4.2. Mesh Model

The models included a finer mesh for the CF-parts because of its importance for the structure performance, and a higher-sized sized mesh for the metallic parts consistent with the very different deformations and rigidities of the parts. Typical mesh smoothing tools, as corrections for mapped surfaces, were used when possible.

The mesh of the tiles was done with 10-node tetrahedral elements. The element size was set to 15 mm with at least three nodes in its thinner zones, resulting in about 14,000 elements per part. Since the ribs geometry is more irregular, a smaller size mesh of 5 mm made of hexahedral elements was used, resulting in about 21,000 elements per part.

For the CF-parts and due to its thin-wall geometry, it is recommended to use shell-elements for better efficiency and adaptation. However, the pre-processing package ANSYS-ACP requires solid elements, and therefore 8-node hexahedral elements of 1 mm in size were initially used, resulting in about 80,000 elements per part. A mesh-sensitive analysis was conducted to determine the adequate size of the elements for these parts also used in other models. The obtained results for a reference model of the setup with element sizes ranging from 1 to 8 mm showed deformation values clearly converging with decreasing element size. The relative differences were below 4.5% in the size ranging between 1 and 4 mm. However, the CPU calculation time increased by a factor seven in that same range. Because of this behaviour, the size value was set to 4 mm as a compromise for accuracy and CPU time, adding uncertainty to the results while assuming a deformation difference below 5%.

For models with (virtual) isotropic CFRP* material, a mesh of 20-node hexahedral elements was used, resulting in 17,350 elements per part.

In Figure 3, there are views of the mesh model used for the setup with two CF-parts. Figure 3a shows the mesh model of the assembly and Figure 3b shows a detail view of the region where two flaps of two ribs are grabbing the CF-part wall.

### 4.3. Model Boundary Conditions: Loads, Support and Contacts

In this subsection, the definitions for the loads, fixed support and contacts of the FE models are described.

**Figure 4:**
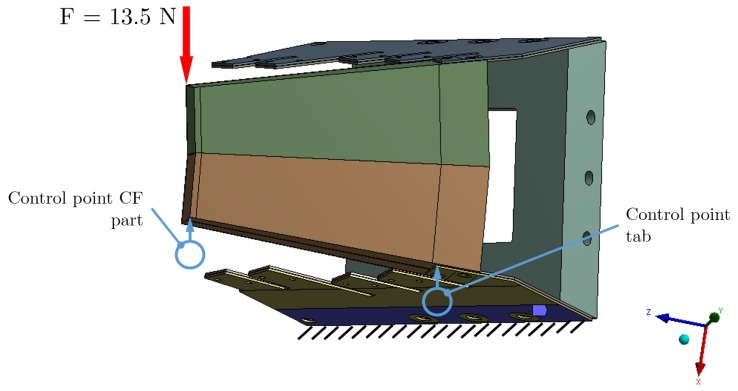
Drawing of the FE model used for the setup with two CF-parts. The point-like loads, fixed support and control points are marked.

**Figure 5:**
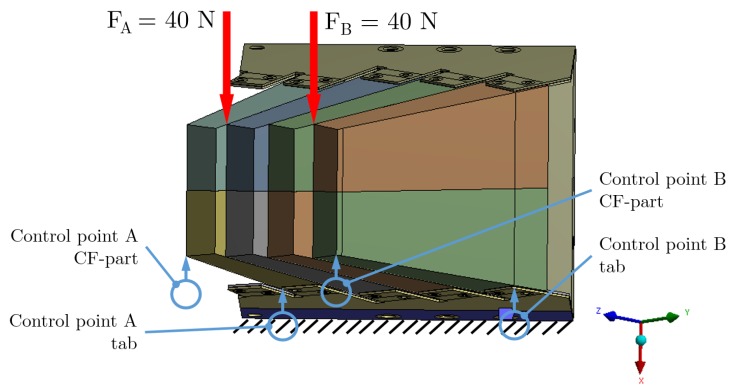
Drawing of the model used for the setup with eight CF-parts.

In the mechanical test setup, the tile was bolted to a bearing support. Therefore, at the rib on the horizontal plane, a fixed strip-like region with width of 20 mm was defined for isostatic balance (Figure 4 and Figure 5). The high rigidity of that region made no difference with respect to the actual mounting with a bolted joint of the tile, ribs, and bearing support (Figure 2, Figure 4 and Figure 5).

The load applied with a *finger-like stick* in the mechanical test setup became a point-like load in the model. For all models, these loads were applied on a reduced diameter region defined in the model; the value, direction, and location had to be as close as possible to the test conditions (Figure 4 and Figure 5).

In the real structure, the actual sensors fill the CF-parts and cause the loading of the whole structure. The different orientations of the sensors cause the loading to lean over any of the CF-part faces depending on the region of the structure. The actual loading of the structure was a challenging problem for the FE model definition because the hundreds of new parts added. The model cannot use approximations such as *remote loads* due to the many orientations of the CF-parts, neither point-like masses instead of sensors due to different position of the centre of mass of CF-part and sensor. Therefore, the sensors and their weight were included in the models.

To study this problem, a real loading test was done cf. Section 3. The *realistic loading* for the two CF-parts setup was defined, where blocks to replace the sensors were added; thus, for the FE model, these blocks and their weight were included.

The study of the contacts [30] was another key for the adaptation of the models. Changes were implemented to relax the contact definitions while keeping the performance of the calculations within known limits as they were provided directly by the reference data.

**Figure 6:**
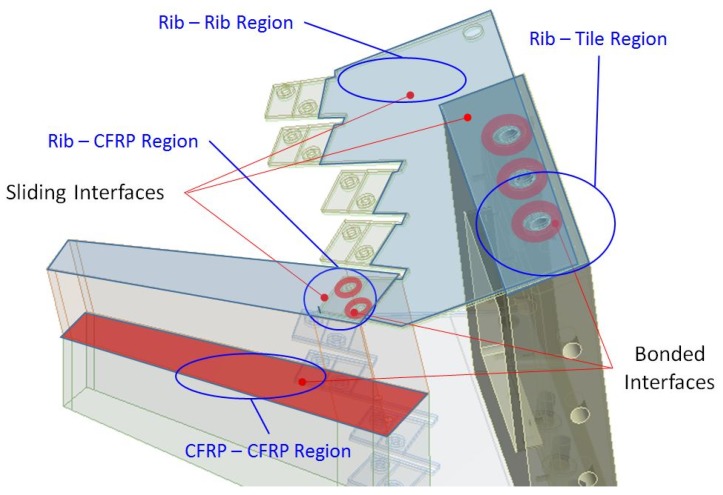
Drawing showing the contact regions at the two CF-parts set up. The pressure cone surfaces are marked, and contact regions indicated.

Three contact regions were first studied: *RIB-RIB*, *RIB-TILE*, and *RIB-CFRP* (Figure 6). These parts were fastened with bolts; therefore, all these regions contain some bolted sub-region. The pressure-cone method was used with a cone-angle value of 45 degrees [31,32] to define the effective fastening region (bonded contact, linear type) which provided the right rigidity of the joint. Beyond the pressure-cone sub-region, models were studied with two types of contact: frictional (nonlinear) contact and with no-separation restriction (linear) contact allowing slipping. All contacts used a penalty-based formulation. Furthermore, frictional contacts follows Coulomb’s friction law in order to define tangential stresses. Special attention was paid for nonlinear models when defining *master* and *slave* surfaces. In that case, the criterion was to define the stiffer surface as the master one, and therefore the CFRP material was defined as slave when in contact with metal parts (rib).

The *RIB-RIB* and *RIB-TILE* regions showed equivalent results with both contact types; this behaviour was most likely caused by the high rigidity of those regions, including only the metal parts. The contact type selected for these regions was the (linear) no-separation restriction. Then, to further study the possible linearization of the contacts, the focus was set to analyze the *RIB-CFRP* region, which was also fastened with bolts at the flaps. Different models were defined with linear and nonlinear contact types to analyse the different effects. The coefficient of friction between CFRP and the steel was set according to [33].

For the two CF-parts model, two load cases were studied, one with *point-like loads* and other with *realistic loads*. In the second case, a metal block was inserted inside each CF-part, which generated new contact regions. In the real case, even though the CF-structure held the sensors inside, these ones must be free and take no loads due to their fragility, and also to avoid compromising their integrity. In the model, to avoid these stiffening effects in the structure when adding blocks, the sensor mounting conditions were emulated by setting the material rigidity (Young’s modulus) of the block to a negligible value. The contact *CFRP-BLOCK* was defined as a bonded (linear) type. The interplay of the definition of this contact and the rigidity is discussed in Section 5.1.

The CF-parts were glued together with epoxy resin and, to avoid including a complex glue-like contact with extra glue material between the CFRP walls, a bonded contact was used instead at the *CFRP-CFRP* region (Figure 6). However, for the model with eight CF-parts, this contact resulted in an extremely stiff structure. To better describe the wall gluing effect, the formulation of the bonded contact was done with control parameters for tangential and normal contact stiffness. This option is a possible approach to tackle contact problems [34], and it is available with ANSYS. Both tangential (FKT) and normal (FKN) control factors of this formulation can be set to values ranging from 0.01 to 1.0. Since the factors only tuned the bonded nature, the contact itself was kept. This procedure allowed for smoothing the bonded character of the contact, and the FKT and FKN values were set according to the results obtained in the models as compared with the test values. The factors of the contact must be common for both test cases, with two and eight CF-parts; however, and due to the rather small glued surface in the two CF-parts case, the gluing effect is limited. The minimum recommended value of 0.01 was used for FKN, and caused no material penetration issues in any setup. For the FKT values, the range from 0.1 to 1.0 caused no appreciable differences when applied to the models for two CF-parts, and, therefore, the value of 0.1 for FKT was set for all models.

## 5. Results and Discussion for Mechanical Test Setups and FE Models

After defining the mechanical test setups and the configuration of the FE models, the results of the models were analysed and compared to the reference data to find a balance of a robust and simplified model still capable of providing realistic values of deformation and within a known uncertainty.

It is important to note that, in this work, only deformation values are presented and discussed. The reason for focusing on deformation is based on the immediate influence on the sensor position and the ultimate sensitivity of the calorimeter. From the mechanical design point of view, the stress is also a key parameter that has to be evaluated and considered to make any design decision, being directly related to safety concerns. A similar discussion as the one presented can be made to assess the stress conditions. However, it is beyond the scope of this work. It is only mentioned that the stress values obtained from the models were coherent with changes, and always far below any stress limits for safety.

### 5.1. Models with Two CF-Parts

In Figure 4, the drawing of the two CF-parts model is shown. The bearing region is defined as fixed at the horizontal (YZ) plane; the applied point-like load marked with a vertical red arrow is located at the upper corner of the CF-part, and the two control points in blue are positioned at the corners of the CF-part and the rib.

#### 5.1.1. FE Model-1: CFRP Pre-Processing and Nonlinear Contacts

The reference model for this setup was defined with the pre-processing of the orthotropic material for the CF-parts and the nonlinear type contacts As explained in Section 4.3, for the *RIB-RIB* and *RIB-TILE* regions, the selection of no-separation (linear) or frictional (nonlinear) contact types caused no differences at this point. However, this model used a realistic frictional contact type at the *CFRP-RIB* region beyond the pressure cone of the bolts.

The values obtained in both control points resulted in being very similar to those obtained in the mechanical test setup, cf. Model-1 in Table 3, corroborating that both the FE model definition (including mesh and contacts) as well as the CFRP material characterization were highly reliable and provided robust results for further discussions.

#### 5.1.2. FE Model-2: CFRP* Isotropic Material and Nonlinear Contacts

Due to the high modelling time for the CFRP pre-processing, the possibility to re-define the CFRP as a (virtual) isotropic material was studied, considering that the fabrics and layers used would result in three-axial symmetric properties. As cited in Section 4.1, the CFRP material was redefined as a (virtual) isotropic CFRP* (cf. Table 2). The same set of contacts as in Model-1 was used, and the results showed a fair agreement at the CFRP corner control point (8% deviation relative to the measured value) and within the uncertainty. However, the result at the rib control point was poor when using the new material, which reduced the rigidity at that flap region.

#### 5.1.3. FE Model-3: CFRP* Isotropic Material and Linear Contacts

The next step of the study was focused on the contact type at the *CFRP-RIB* region and, to simplify the calculation time, the contact was defined as a no-separation (linear) type. This modification is expected to induce a higher rigidity in the structure and counterbalance the low rigidity of Model-2 due to the material re-definition. Additionally, the new model was the most simplified due to the full linearization of all the contact types, including the isotropic character of the virtual material CFRP*.

The results at the CFRP corner control point differed by 21%, and at the flap control point resulted in being very similar to the measured value. Therefore, the deviations were proportional to the distance between the control points and the bearing region. As expected, the trade-off of effects of the combined approximations for material and contact definitions resulted in being effective since the functional description of the FE model was kept even though it was rather simplified.

#### 5.1.4. FE Model-4: CFRP* Isotropic Material and Realistic Loads

By comparing the results, it can be stated that the FE Model-3, including the properties of the (virtual) isotropic CFRP* material, as well as linear-type contacts between the parts, provides reliable results of deformations within the uncertainty limit of 21% at the point of maximum deflection of the CF-part located away from the bearing regions.

Based on Model-3, a new FE model was studied, where the type of applied loads was changed to the weight caused by sensor-like blocks. As previously mentioned in Section 4.3, when changing to realistic loads, a new contact region arose. This new contact *CFRP-BLOCK* was defined as a bonded (linear) contact type. The use of this contact for the CFRP-block region raised the question of a possible deformation of the CF-part walls because of the combination of a low-rigidity value assigned to the block (cf. Section 4.3) and the thin-walls of the CF-parts. An independent evaluation of the *CFRP-BLOCK* contact was defined as a realistic frictional contact and the actual rigidity of the material provided wall deformation values rather similar (below 7% deviation) to the selection presented here. More importantly, the iso-surfaces of the stress and deformation distributions showed the same pattern and had comparable values for both models. Therefore, the linear option was selected as the most adequate for our purposes.

The results provided by this model are listed in Table 4. The displacement results are similar to the obtained in the mechanical test setup within 6% deviation at the rib control point and 9% deviation at the CFRP corner control point.

The use of distributed loads resulted in a better performance of the model. Even though the reference Model-1 under point-like load resulted in being very accurate, the CFRP pre-processing causes limitations to deploy to many parts’ structures. On the other hand, Model-3 with its distributed (realistic) load counterbalanced the limitations caused by modifying the contact character, and therefore it was more adequate for the description of the whole structure.

### 5.2. Model with Eight CF-Parts

The eight CF-parts model allowed us to perform the study of the effects produced by increasing size and number of parts in the FE model, as well as the evaluation of these scaling effects for the whole structure. For the eight CF-parts model, a point-like load was applied (Figure 2b and Figure 5). The bearing region located at the horizontal plane (YZ) was defined as fixed. At the edge between two parts and away from the bearing region, point-like loads (red vertical arrow) were applied at two different positions (*A* and *B*) for independent tests with four control points (marked in blue) at the corners of the CF-parts and the rib located at the horizontal plane.

The FE model for this setup was done according the Model-3 definitions for the 2 CF-parts setup. The most important differences in this model were the large regions with *CFRP-CFRP* contact caused by the gluing of all the CF-parts, and defined as discussed in Section 4.3. The deformation values were calculated and compared with the measured data for the two independent load test at points *A* and *B* (see Table 5). The values differed at the rib point ranging from 0 to 21% (relative values), and for the CFRP corners ranging from 48 to 53%. The models always provided lower deformation values corresponding to a more rigid structure than the real one. This stiffening effect was observed in Model-3 (cf. Table 3), with a deviation of 21%, as well as in the model with the realistic load (Table 4), which had a deviation of 9%, both referring to the control point at the CF corner. Therefore, this stiffening effect was mostly due to the material and contact selected, and was in no case caused by the large glue-contact. Setting the *CFRP-CFRP* contact parameters (FKT and FKN) to negligible values did not notably change the results and, consequently, demonstrated the robustness of the model to this tuning selection.

Looking only at the load point at side *B*, the results of the setups with two and eight CF-parts (Table 3 and Table 5) can be compared. The deformation, within the uncertainty, was similar in both cases even though, in the second case, the load applied was three times higher. Clearly and as expected, when more parts were added to form the structure, the stiffer it became. Moreover, when the load was applied at side *A*, the deformation values obtained were similar to those in the case where the load was applied at side *B* for opposite points; therefore, these results showed that both the model and the setup were highly symmetrical despite the real geometry.

## 6. FE Model for the Instrument Structure

**Figure 7:**
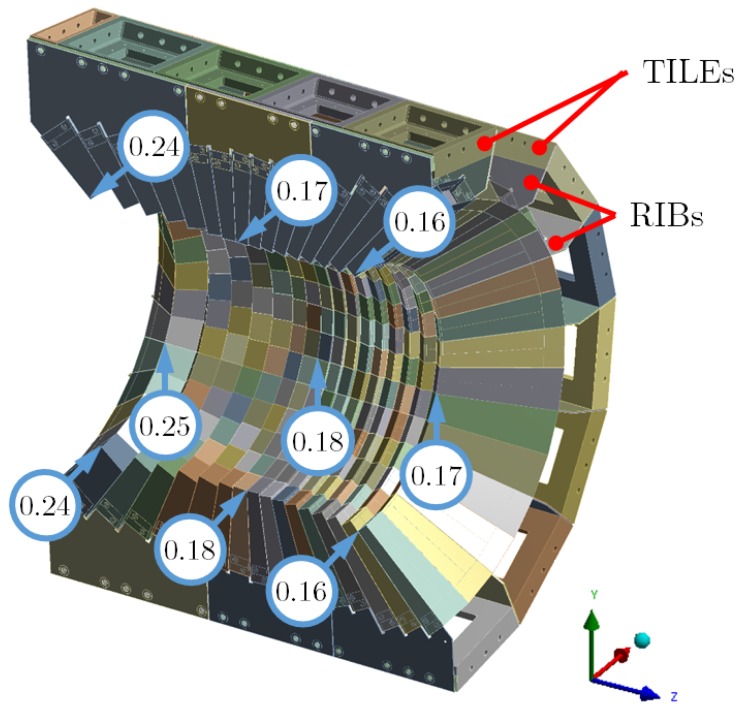
Drawing of the structure showing the maximum nodal displacements at the corners of the CF-structure.

After studying all the selections applied to the FE models previously mentioned in the sections above, the main objective of this research can be tackled by evaluating an FE model for the calorimeter structure as a whole, considering just one half of the structure due to its symmetry at the vertical mid-plane (Figure 1d and Figure 7).

The material was defined as CFRP*; the contacts were defined as bonded type at the pressure cones for the bolted joints, tuned bonded types for the CFRP–CFRP regions and non-separating type in any other region; the sensors weight were defined as the applied load. The support of the system was also defined as if the cover was fixed at some faces to an external frame [14]. The results of the model calculations showed a negligible deformation (well below 0.01 mm) of the stiff cover structure parts. Therefore, for the evaluation of the structure design, the points with the maximum deflection were selected, which were the nodal points located at the corners of the CF-parts in the internal opening and away from the bearing region. In Figure 7), the values calculated for different corners of the CF-structure are shown. The points correspond to the upper and bottom positions at the mid-vertical plane and the mid-horizontal plane.

Then, taking into account the maximum deformations in the CF-structure, the reliability, modelling, and computing time for the whole structure were determined.

### 6.1. Model Deformations

The deformation values at the nodal points were rather similar in each half-ring, the variation being just a few micrometers along the same half-ring. The values were slightly higher at the lower regions with respect to the upper one. This difference was caused by the cumulative effect of the weight acting from top-to-bottom at each half-ring. The tiny differences show the capability of the honeycomb structure to provide stiffness in every region of the CF-structure.

The deformation values also changed along the longitudinal axis from 0.16 up to 0.24 mm (Figure 7). The different position of the rib-flaps relative to the bearing region at the tiles changed the lever arm and caused the different deformation along the axis.

The tri-axial deformation at the CF corner points determined in which direction the structure was deflected and which region was affected. Along each half-ring, the deformation changes direction from top to bottom: at the upper region, the deflection was mostly horizontal and in the positive *x*-axis direction; at the mid-horizontal plane, the deformation was in the vertical direction and downwards (negative *y*-axis); and, at the lower region, the deflection was again horizontal but pointing to the negative *x*-axis direction. These directions are expected for the loading caused by the weight. This effect combined to the axial difference, which makes a torsional-like deformation in the CF-structure. The high rigidity of the CFRP structure made the deformation very limited in value at the most unfavorable locations.

### 6.2. Model Reliability

For the model calculations, the comparison with the measured data defined the uncertainty, ranging from 9 to 53% for the most critical points, the CFRP corners far away from the bearing regions. All these depended on the load cases, where the lower value corresponded to the realistic load distribution, and also depended on the type of structure with either two or eight CF-parts.

Cumulative uncertainties for the whole structure were tough to trace back. On the one hand, the stiffening effect inherited in the model grew faster in the model than in the mechanical setup, with the increasing number of parts. It showed deviations up to 21% and 53% for two and eight CF-parts, respectively (cf. Table 3 and Table 5). On the other hand, the use of a realistic load distribution resulted in a difference reduced by a factor 2.3 (21% to 9% deviation, cf. Table 3 and Table 4). This effect was not expected to be related to the structure size, but to the load nature. Due to the addition of parts and the application of real load distributions, and based on the previous discussion, a sound deviation up to 370% was estimated as possible. This evaluation gives a rough estimated maximum deformation value of 0.9 mm at the CFRP corner points.

To have a better picture of the quality of these results, the uncertainty linked to the production of the parts, the bundles of parts and the mounting of the whole structure has to be considered [15]. The inner volume of the parts was obtained with tolerances of 0.05 mm in any plane; however, the outer surfaces and edges were not so tightly constrained. Additionally, the CF-parts were glued together in a full handcraft process. The measured fluctuations in envelope dimensions of eight CF-parts reached up to 1 mm, and similar values are typical for mounting the parts into the cover structure. According to the FE model and assuming maximal uncertainties, the expected deformation values (0.9 mm) remained well below the added tolerance values of production and assembly added together (2 mm). This situation showed that the model evaluation was properly suited to provide design decisions, even considering the largest uncertainties.

### 6.3. Modelling and Computing Time

One of the key goals for the development of this model was the possibility to have an acceptable modelling and computing time. Complex mechanical calculations tough to define and containing thousands of features are hardly useful to explore design options in an effective way during the timeline of typical projects. In our model, the use of a material pre-processor (a critical model definition step) was avoided, and only linear contact sets were used, guaranteeing the convergence despite the large number of features.

The time involved in the pre- and post-processing stages of the model was evaluated to be between 20 and 25 h, using ANSYS or similar software packages, which typically included *boosting* tools, e.g., to define symmetrically located contacts. The calculation took 38.3 h based on a single CPU IntelCore i7-4720HQ (Intel Corporation, Santa Clara, CA 95054-1549, USA), 3.60 GHz, 16 Gb RAM, and ANSYS v15 (64 bits). The total time is fully compatible with a 10-day time frame dedicated to mechanical calculations, including the model definition, revision, evaluation, and analysis.

## 7. Conclusions

This work analysed a case study involving initially a highly nonlinear FE model for a complex thin-wall structure made of CFRP materials with the goal to make an FE model robust and reliable enough as to be used as a design tool for the study stage of one scientific instrument. The complexity of the FE model arising from the many parts, features, and details was a tough bottleneck for implementing such FE model to work in a flexible and effective way and provide the right feedback to the rest of the project stages.

For the validation of the FE model, two mechanical setups, one of two CF-parts and the other with eight CF-parts, were mounted. The FE models studied allowed for selecting the right set of contact definitions and the CFRP properties as (virtual) isotropic material, so that the displacement results obtained were within 9% deviation for a realistic load.

With these considerations, a full linear model was successfully defined with sound assumptions, capable of reproducing the results within known deviations, and, once extended to the whole structure, yielded the next results:

- The overall time necessary for the model definition and calculation fits in a reasonable time span to interact with the project needs, despite the large amount of parts and contacts defined in the FE model.
- The total deformation calculated provided values below 0.24 mm in the key regions. The overall uncertainty expected from small setups to the whole structure was estimated and a sound maximal deformation was set (0.9 mm). For comparison, the production and mounting tolerance values remain much higher (220% factor). Therefore, the FE model can definitely provide useful results for the design project.

The kind of questions tackled down with this FE model were design proposals raised through the project process, including large volume changes of CF-parts, CFRP wall thickness limits, CF-part shapes, etc. [15]. The methodology developed can be deployed to other cases demanding intensive mechanical evaluations of complex (many parts) structures including CFRP materials.

## Figures and Tables

**Table 1:** Mechanical properties of the CFRP fabric as determined according to the micromechanical model in [27]: Young’s modulus (E), shear modulus (G) and Poisson’s ratio (ν). *x* and *y*-axis directions are in-plane. *z*-axis direction is out-of-plane (layer thickness).

Density [g/cm^3^]	$E_{x}$,$E_{y}$ [GPa]	$E_{z}$ [GPa]	$G_{\mathrm{xy}}$ [GPa]	$G_{\mathrm{yz}}$,$G_{\mathrm{xz}}$ [GPa]	$\nu_{\mathrm{xy}}$	$\nu_{\mathrm{yz}}$,$\nu_{\mathrm{xz}}$
1.48	57.98	12.06	4.18	3.81	0.05	0.44

**Table 2:** Mechanical properties of the isotropic materials used in the models.

	Density [g/cm^3^]	Young’s Modulus [GPa]	Shear Modulus [GPa]	Poisson’s Ratio
Aluminium 5083	2.67	71.0	26.69	0.33
Steel AISI 304	8.0	193.0	74.81	0.29
CFRP*	1.48	57.98	22.30	0.30

**Table 3:** Measured and model results for the setup with two CF-parts and a point-like load.

	CFRP	Contact	Deflection	Deflection
	Pre-Process	CFRP-RIB	Rib-Flap [mm]	CF-Corner [mm]
test			0.065	0.140
model -1	yes	nonlinear	0.069	0.142
model -2	no	nonlinear	0.119	0.151
model -3	no	linear	0.066	0.110

**Table 4:** Data and results of the model for the setup with two CF-parts and sensor-like blocks.

	CFRP	Contact	Deflection	Deflection
	Pre-Process	CFRP-RIB	Rib-Flap [mm]	CF-Corner [mm]
test			0.266	0.558
model-4	no	linear	0.283	0.505

**Table 5:** Data and results of the model for the setup with eight CF-parts and point-like loads.

	Load Point	Pre-Process	Contact CFRP-RIB	Deflection Rib-Flap		Deflection CF-Corner	
				A [mm]	B [mm]	A [mm]	B [mm]
test	A			0.070	0.040	0.160	0.100
model	A	no	linear	0.058	0.040	0.076	0.052
test	B			0.050	0.070	0.120	0.130
model	B	no	linear	0.043	0.055	0.055	0.066

## Abbreviations

The following abbreviations are used in this manuscript:

FE	finite element
CFRP	carbon-fibre reinforced plastic
CF	carbon fiber
CFRP*	(virtual) isotropic material
FKT	bonded contact stiffness factor, tangential component
FKN	bonded contact stiffness factor, normal component
RIB-TILE	contact region of the rib and tile parts
RIB-RIB	contact region of two rib parts
RIB-CFRP	contact region of the rib and CFRP parts
CFRP-BLOCK	contact region of the CFRP and block parts
CFRP-CFRP	contact region of two CFRP parts
